# Plant Growth-Promoting Bacteria (PGPB) integrated phytotechnology: A sustainable approach for remediation of marginal lands

**DOI:** 10.3389/fpls.2022.999866

**Published:** 2022-10-21

**Authors:** Vikram Poria, Klaudia Dębiec-Andrzejewska, Angelika Fiodor, Marharyta Lyzohub, Nur Ajijah, Surender Singh, Kumar Pranaw

**Affiliations:** ^1^ Department of Microbiology, Central University of Haryana, Mahendergarh, India; ^2^ Department of Environmental Microbiology and Biotechnology, Institute of Microbiology, Faculty of Biology, University of Warsaw, Warsaw, Poland

**Keywords:** phytoremediation, plant growth-promoting bacteria (PGPB), marginal land, biodegradation, heavy metals (HMs), organic pollutants

## Abstract

Land that has little to no utility for agriculture or industry is considered marginal land. This kind of terrain is frequently found on the edge of deserts or other arid regions. The amount of land that can be used for agriculture continues to be constrained by increasing desertification, which is being caused by climate change and the deterioration of agriculturally marginal areas. Plants and associated microorganisms are used to remediate and enhance the soil quality of marginal land. They represent a low-cost and usually long-term solution for restoring soil fertility. Among various phytoremediation processes (viz., phytodegradation, phytoextraction, phytostabilization, phytovolatilization, phytofiltration, phytostimulation, and phytodesalination), the employment of a specific mechanism is determined by the state of the soil, the presence and concentration of contaminants, and the plant species involved. This review focuses on the key economically important plants used for phytoremediation, as well as the challenges to plant growth and phytoremediation capability with emphasis on the advantages and limits of plant growth in marginal land soil. Plant growth-promoting bacteria (PGPB) boost plant development and promote soil bioremediation by secreting a variety of metabolites and hormones, through nitrogen fixation, and by increasing other nutrients’ bioavailability through mineral solubilization. This review also emphasizes the role of PGPB under different abiotic stresses, including heavy-metal-contaminated land, high salinity environments, and organic contaminants. In our opinion, the improved soil fertility of marginal lands using PGPB with economically significant plants (e.g., *Miscanthus*) in dual precession technology will result in the reclamation of general agriculture as well as the restoration of native vegetation.

## 1 Introduction

Marginal lands are increasing at an alarming rate due to various anthropogenic activities. Marginal lands are defined as having inferior soil with inadequate agricultural attributes and crop yield that is occasionally polluted. The state of the world’s land and water resources for food and agriculture is associated with the condition of our productive land and ecosystems and reflects problematic trends in resource consumption (FAO, 2022). A rapid decline in natural and seminatural ecosystems over the last centuries and severe climatic changes along with increasing human pressure have led to more frequent extreme weather events, higher rates of land degradation, and potential habitat losses. Furthermore, excessive use of fertilizer impacts the health of soil negatively. Thus, remedial actions are needed to prevent land degradation, on which 98% of the world’s food is produced.

The degradation of land, soil, and water resources as a result of human activity diminishes their production potential, biodiversity, and environmental services that support healthy and resilient livelihoods. Energy crops planted in these regions can aid in land reclamation and significantly reduce greenhouse gas emissions (Zhuang et al., 2011; Schröder et al., 2018). Overgrazing (35%), intensive agriculture (28%), deforestation (30%), manufacturing fuel wood (7%), and industrialization (4%) are the main drivers of soil deterioration (Schröder et al., 2018). Land might be marginal for a variety of reasons, including a lack of water supply, low chemical and/or microbiological soil quality, pollution from previous industrial activity, topographic obstacles such as an extreme slope, or when inaccessible or very remote from transportation networks. Most marginal lands are also characterized by heavy metal (HM) contamination, organic pollutants, strong acidification or alkalization, high salinity, limited water, etc. (Jiang et al., 2021; Shahane and Shivay, 2021). According to Fan et al. (2020), for energy crop production, about 43.75 million hectares (Mha) of marginal land is available in South China, 11.36 Mha in the US, 1.4 Mha in the UK, and almost 45 Mha in Europe. On the other hand, Jiang et al. (2021) assessed the total marginal land worldwide suitable for the cultivation of the energy crop *Pistacia chinensis* using a machine learning method and reported that a total of 1311.85 Mha of marginal land is mostly distributed in Southern Africa, the southern part of North America, the western part of South America, Southeast Asia, Southern Europe, and the east and southwest coasts of Oceania.

Plant growth is adversely affected by increased salinity or HM concentration, too high or low pH, low water availability, and the presence of other contaminants. Wilting and abscission of leaves, decreased leaf regions, and decreased water loss through transpiration are all physiological responses to stress in plants. Reduced turgor pressure under stress is one of the most delicate physiological mechanisms that allows cells to develop in a stressed environment. Drought stress disrupts water passage from the xylem to surrounding elongating cells in higher plants resulting in cell elongation suppression. In addition, drought leads to a reduction in leaf area, plant height, and plant development as cell elongation, mitosis, and spreading are impaired. Osmotic modification, which supports the active accumulation of solutes in the cytoplasm to maintain the cell’s water balance, can reduce the negative effects of stress (Yadav et al., 2020).

Plants employ various mechanisms, such as escape, avoidance, and tolerance, to counteract various stresses, and many plants have been described for their soil remediation ability. Plants can remove or immobilize various pollutants from the soil by employing different phytoremediation strategies, such as phytodegradation, phytoextraction, phytostabilization, etc. (Yan et al., 2020). PGPB also helps to improve the remediation ability of plants by stimulating plant growth through the secretion of various types of metabolites and hormones, solubilizing minerals, fixing nitrogen, and protecting plants against pathogens. PGPB also helps in alleviating various other types of biotic and abiotic stresses faced by plants (Backer et al., 2018). In this review, various phytoremediation strategies and the role of PGPB in enhancing the plant’s potential for phytoremediation have been discussed. In addition, a number of plant growth-promoting activities of PGPB have also been reported.

## 2 Phytoremediation

Phytoremediation is a decontamination technique wherein plants, such as grasses, shrubs, and trees, help to clean up the environment by degrading, accumulating, or stabilizing the contaminants. Phytoremediation techniques generate very little secondary waste while removing contaminants from the environment in an environmentally friendly way and at a very low cost (Shah and Daverey, 2020).

### 2.1 Types of phytoremediation and their mechanisms

**Figure 1:**
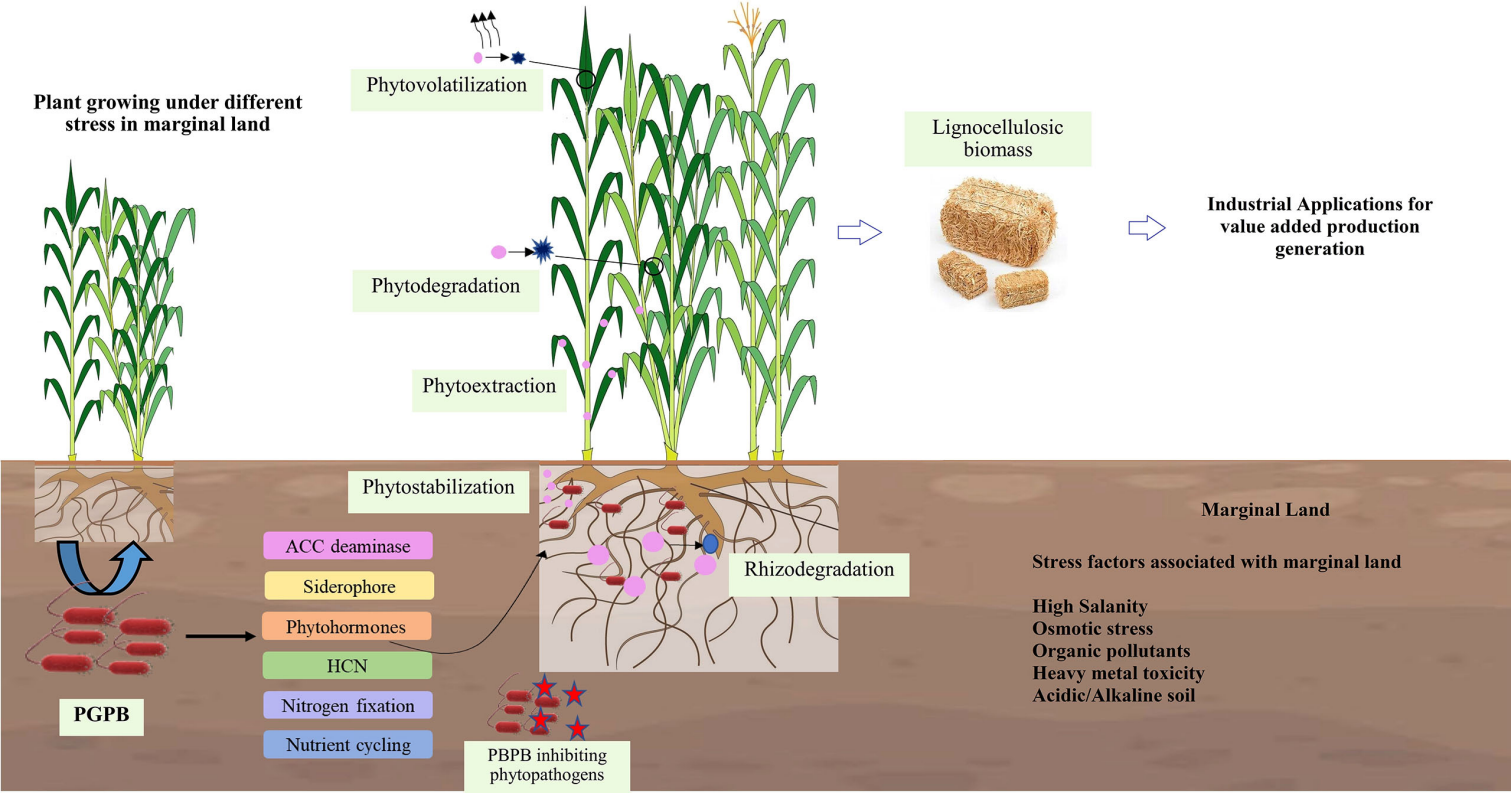
Phytoremediation methods and the role of PGPB in assisting plants for the remediation of marginal lands.

Based on the soil conditions, pollutants, and plant species, phytoremediation of HM-contaminated soils includes many methods and processes such as phytodegradation (phytotransformation), phytofiltration, phytoextraction (phytoaccumulation), phytostabilization, rhizodegradation, phytodesalination, and phytovolatilization, as shown in **Figure 1** (Saleem et al., 2020b; Nedjimi, 2021; Sabreena et al., 2022). The most extensively employed phytoremediation methods in the remediation of HM-contaminated soils are phytostabilization, phytoextraction, phytovolatilization, and phytofiltration, whereas strategies like phytodegradation and rhizodegradation are used for the degradation of organic pollutants (Yan et al., 2020). The effectiveness of phytoremediation depends primarily on the plant species used for contaminant removal. Plant species with high metal tolerance and high extraction or immobilization ability are very effective and are usually used for phytoremediation (Visconti et al., 2022). There are several newly emerged phytoremediation techniques such as phytoremediation buffers, phytoremediation vegetation, phytoremediation caps, phytoremediation plantings, and percolation and phytoremediation (Riaz et al., 2022).

#### 2.1.1 Phytostabilization or Phytoimmobilization

Phytostabilization is a cost-effective and less invasive phytotechnology that stabilizes hazardous contaminants such as HMs in the rhizosphere or roots by using tolerant plants that bind the contaminants in soil reducing their mobility within ecosystems and food chains (Shackira and Puthur, 2019). It involves a temporary accumulation of contaminants by plants in their belowground parts, which only immobilize and deactivate toxic metal ions instead of permanently removing them from the contaminated soil by forming metal complexes with root exudates, cell wall bonds, precipitates, or reducing HMs in the rhizosphere, which are sequestered in the vacuoles of root cells with the help of microorganisms (Zgorelec et al., 2020; Kafle et al., 2022). For effective phytostabilization, the selection of suitable plant species is very important. The plant should be HM tolerant and have a dense root system because roots play an important role in HM immobilization, stabilizing the soil structure, and preventing soil erosion (Shackira and Puthur, 2019; Zine et al., 2020). In addition, plant species and varieties characterized by low translocation factors are also highly desirable (Huang et al., 2021).

#### 2.1.2 Phytodegradation

Phytodegradation is the degradation of contaminants taken up by plants through metabolic processes, or the degradation of pollutants outside the plant by enzymes (such as dehalogenases, nitro reductases, and peroxidases) released by roots (Jhilta et al., 2021; Nedjimi, 2021). Rhizosphere microorganisms perform most of the phytodegradation activities in a favorable rhizosphere environment. The ability of plants to modify the rhizospheric microbial community composition (Poria et al., 2021b) and microbial interaction within it has a significant impact on the degradation of pollutants. There are many reports which suggest that plants promote the degradation of contaminants by rhizospheric effects (Kotoky and Pandey, 2020a; Kotoky and Pandey, 2020b; Nebeská et al., 2021).

#### 2.1.3 Phytoextraction or phytoaccumulation

Contaminants from soil or water are taken up by plants and permanently accumulated in above-ground biomass through translocation during phytoextraction or phytoaccumulation (Ali et al., 2020). Phytoextraction involves several steps, starting with HM mobilization in the rhizosphere, followed by uptake by plant roots and translocation to above-ground parts of the plant. The final step is the immobilization and compartmentalization of HM ions in the aerial tissues of the plant (Yan et al., 2020).

#### 2.1.4 Phytovolatilization

In this phytoremediation strategy, plants release volatile forms of HMs by transpiration through the leaves after absorbing HMs from the soil and converting them to less toxic volatile forms (Bortoloti and Baron, 2022). Compared to other phytoremediation strategies, phytovolatilization is more beneficial as it removes contaminants without harvesting and disposing of the plants and converts them to gaseous compounds (Yan et al., 2020).

#### 2.1.5 Phytofiltration

In this strategy, reclamation of soil and water with a low content of contaminants is carried out using seedlings (blastofiltration), shoots (caulofiltration), or roots (rhizofiltration). In rhizofiltration, HMs are either adsorbed on the root surface or absorbed by the roots. Root exudates can alter the pH of rhizosphere, resulting in HM precipitation at plant roots and a reduction in HM transport to subsurface water. Plants used for rhizofiltration are first cultivated in clean water (hydroponics) to establish an extensive root system, and then contaminated water is utilized to acclimate the plants. These plants are then transferred to the contaminated site for HM removal. The roots are removed and discarded once they become soaked with HMs (Ashraf et al., 2019).

#### 2.1.6 Rhizodegradation or phytostimulation

Rhizodegradation is the biodegradation of organic pollutants in the soil, which involves the secretion of certain enzymes by rhizospheric microorganisms that degrade or convert heavily polluted organic pollutants into less harmful substances. The rhizodegradation process is accelerated by microbes that take up nutrients (amino acids, carbohydrates, etc.) from the root secretions of the plant, increasing its efficiency and accelerating the extraction and removal of contaminants (Ashraf et al., 2019; Sabreena et al., 2022). Dissolution of the pollutant at the source is a key element of rhizodegradation, which focuses on the complete mineralization of the organic pollutant by transferring it to the plant or atmosphere. Soil type and plant species has an effect on rhizodegradation. *Bacillus*, *Burkholderia*, *Pseudomonas*, *Agrobacterium*, *Alcaligenes*, *Arthrobactor*, and *Micrococcus* are bacteria frequently reported to be involved in rhizodegradation, as are mycorrhizal fungi such as *Glomus* (P.P and Puthur, 2021).

#### 2.1.7 Phytodesalination

Phytodesalination is the most widely used method to decontaminate saline soils and uses halophytic plants. In comparison to other phytoremediation approaches, knowledge about this strategy is still poor. Halophytes are thought to be naturally well-adapted to live in HM- contaminated surroundings in contrast to most glycophyte plants. The plant’s ability to desalinate soil is determined by the species, as well as soil properties like salinity, sodicity, and porosity, as well as other climatic elements like rainfall (Sabreena et al., 2022).

Several new sustainable phytoremediation techniques are also being used for soil remediation. Such a technique can be used to remediate areas like brownfields, mine wastes, and landfill trash that have low to moderate levels of contamination. Known as phytoremediation buffers, the technique uses buffer strips in conjunction with riparian corridors where appropriate plant species are planted in the stripes along stream channels to remove toxins from surface water and groundwater flowing into rivers. This method is incredibly useful in preventing the further spread and mixing of water pollutants into groundwater supplies. Phytoremediation can result in contaminants entering the food chain, and some phytoremediation techniques do not completely remove contaminants from the environment. To reduce this risk of environmental and food contamination, another combined technique, percolation, along with phytoremediation, can be used to remove and percolate contaminants from the polluted soils. Using infiltration and percolation through vegetative covers, this technique is designed to remove soil pollutants. Phytoremediation caps also serve the purpose of reducing contaminant input to natural resources by reducing infiltration and precipitation and improving cap integrity. To reduce the spread of contaminants from their active hotspots, phytoremediation vegetation cover systems are used to establish barriers and prevent the further spread of contaminants. This is an economically feasible system because it is self-regenerating and can control soil erosion (Riaz et al., 2022)

### 2.2 Major economically important plants used for phytoremediation

Phytoremediation is a cost-effective and ecologically sound method of removing contaminants from soil and water. Adsorption, transport and translocation, hyperaccumulation or transformation, and mineralization are all processes that plants use to remove HMs from the environment (Pandey et al., 2015). Phytoremediation has already been documented for *Festuca arundinacea*, *Hordeum vulgare*, *Thlaspi caerulescens*, *Linum usitatissimum*, *Pteris vittata*, and *Brassica juncea*, among other economically important plants (Saleem et al., 2020a). Marginal lands are suitable for the growth of numerous energy crops, such as *Arundo donax*, *Brassica juncea*, *Jatropha curcas*, *Miscanthus species*, *Ricinus communis*, and *Salix* spp., which have been used to remediate polluted areas (P.P and Puthur, 2021).

Plants with a high degree of ecological adaptability and economic value are selected for their phytoremediation capacity so that, in addition to removing contaminants, they offer other social, environmental, and economic prospects, such as carbon sequestration, increasing soil’s organic biomass, promoting microbial biodiversity, biomass for value-added products such as bioenergy production, platform chemicals, fragrances and flavorings, construction and soundproofing materials for houses, and raw material for the paper pulp industry, etc. These plants, which include *Saccharum munja*, *Saccharum spontaneum*, *Vetiveria zizanioides*, *Cymbopogon flexuosus*, *Ocimum basilicum*, *Ricinus communis*, *Jatropha curcas*, *Miscanthus giganteus* (Pandey et al., 2015), *Cyperus alternifolius*, *Amaranthus retroflexus*, *Celosia cristata*, and *Bambusa vulgaris*, have been reported for their bioremediation and phytodesalination activity (Ameri Siahouei et al., 2020). A list of some economically important plants for phytoremediation is presented in **Table 1**.

**Table 1:** A list of economically significant plants for phytoremediation.

Plant name	Purpose	Contaminant removed	Phytoremediation strategy	Reference
*Amorpha fruticosa*	Phytoremediation ability, N_2_-fixation, stress-tolerant, and tap root system	Pb and Zn	Phytostabilization	Sikdar et al. (2020)
*Helianthus petiolaris*	Bioenergy crop and phytoremediation ability	Pb and Cd	Phytostabilization	Saran et al. (2020a)
*Thymus kotschyanus*, *Phleum pratense*, and *Achillea millefolium*	Phytoremediation ability	Cu	Phytoextraction and phytostabilization	Ghazaryan et al. (2018)
*Atriplex nummularia*	Phytoremediation and stress tolerating ability	Zn, Cu, Cd, and Pb	Phytostabilization	Eissa (2019)
*Pennisetum purpureum* cv. Mott	Bioenergy crop and phytoremediation ability	As and Mn	Phytostabilization	Kowitwiwat and Sampanpanish (2020)
*Azolla filiculoides*	Phytoremediation ability and N_2_-fixation	Sodium dodecyl benzene sulfonate	Phytodegradation	Masoudian et al. (2020)
*Andropogon tectorum*	Phytoremediation ability	Petroleum hydrocarbon	Phytodegradation	Jude et al. (2019)
*Acacia farnesiana*	Bioenergy crop and phytoremediation ability	Crude oil	Phytodegradation	Ahmed et al. (2021)
*Calophyllum brasiliense, Hymenaea courbaril*	Phytoremediation ability	Hexazinone	Phytodegradation and Rhizodegradation	Dos Santos et al. (2018)
*Salix interior* and *Trifolium pratense*	Phytoremediation ability and N_2_-fixation	As, Cu, Cr, and pentachlorophenol	Phytoextraction	Lachapelle et al. (2021)
Cotton	Bioenergy crop and phytoremediation ability	Cd	Phytoextraction	Ramana et al. (2021)
*Linum usitatissimum*	Bioenergy crop and phytoremediation ability	Cu	Phytoextraction	Saleem et al. (2020c)
*Sedum plumbizincicola*	Phytoremediation ability	Cd	Phytoextraction	Zhou et al. (2020)
*Pteris vittata* L. and *Morus alba* L. or *Pteris vittata* L. and *Broussonetia papyrifera* L.	Bioenergy crop and phytoremediation ability	As, Cd, Pb, and Zn	Phytoextraction	Zeng et al. (2019)
*Pteris multifida*	Bioenergy crop and phytoremediation ability	As, Pb, and Cd	Phytofiltration	Rahman et al. (2018)
*Trifolium alexandrinum* L.	Green manure and phytoremediation ability	Cr, Co, and Ni	Phytofiltration	Abuzaid et al. (2021)
*Helichrysum arenarium*	Phytoremediation ability	Au and Ag	Phytoextraction, phytostabilization, and phytoremediation	Vural and Safari (2022)
*Oryza sativa*	Phytoremediation ability	2,4-dibromophenol (2,4-DBP) and 2,4-dibromoanisole (2,4-DBA)	Phytovolatilization	Zhang et al. (2020)
*Cajanus cajan*	Legume (N_2_-fixation) and phytoremediation ability	Petroleum Oily Sludge	Rhizodegradation	Allamin et al. (2020)
*Sorghum* x *drummondii*	Bioenergy crop and phytoremediation ability	Polycyclic aromatic hydrocarbons	Rhizodegradation	Dominguez et al. (2020)
*Daucus carota* L.	Vegetable and phytoremediation ability	2,2′,4, 4′-tetrabrominated diphenyl ether	Rhizodegradation	Xiang et al. (2018)
*Ipomoea aquatica*, *Alternanthera philoxeroides*, and *Ludwigia adscendens*	Vegetable, fodder, and phytoremediation ability	Sodium	Phytodesalination	Islam et al. (2019)
*Puccinellia nuttalliana* and *Typha latifolia*	Phytoremediation ability	Landfill leachate	Phytodesalination	Xu et al. (2019)
*Triticum aestivum* and *Chenopodium quinoa*	Salinity tolerant and phytoremediation ability	Ca, Mg, Na, K, and Cl	Phytodesalination	Hoseini et al. (2022)

### 2.3 Factors limiting plant growth and phytoremediation potential

Phytoremediation is a potential method for removing contaminants from soil, but it has several drawbacks. Decontamination using plants is a time-consuming process due to its complete dependence upon the type and amount of particular contaminants in soil and the plant variety. The majority of plants with phytoremediation potential are slow growing and have lower biomass, limiting their efficacy. Some pollutants are found in soil in a very tightly bound state, limiting their bioavailability and making mobilization difficult. Because plants are not tolerant to extremely high levels of pollutants, phytoremediation is only successful in low-to- moderately contaminated soil/water. Moreover, other soil properties like pH, clay content, and organic matter also limit phytoremediation efficiency (Jaskulak et al., 2020). Effective phytoremediation also necessitates favorable weather and climatic conditions for plants. Storage, handling, and correct disposal of metal-derived phyto-biomass are also difficult due to the constraints mentioned above. Compaction and composting of phyto-biomass reduce bulk and transportation costs while increasing dissolved metal-organic compound leaching. As a result, a key concern with phytoremediation is the long-term disposal of phyto-biomass (Shah and Daverey, 2020). Because plant roots can only uptake contaminants in their immediate proximity and cannot reach deep layers of soil, phytoremediation has a major drawback: It only removes a fraction of the contaminants from the soil. Furthermore, soil may contain many contaminants at the same time, and the plants used to remediate the soil may not be tolerant or hyperaccumulators for all of the chemicals, rendering the process less effective (Pathak et al., 2020).

These challenges can be overcome by increasing the phytoremediation potential of plants by using various breeding techniques to improve their stress tolerance. Plants contain many genes responsible for stress tolerance to metals and are involved in the uptake and transport of these metals. Breeding techniques transfer these genes to increase the potential of plants to thrive under metal stress conditions. Using these techniques, many resistant varieties have been bred such as Parang 401 (Fe chlorosis resistant rice), BRRI-29, BR-26, and BR-14 (As tolerant commercial rice), Pirsabak 2004 (Pb tolerant wheat variety), Micro-Tom (Cd tolerant tomato), and Strongfield (Cd tolerant wheat variety). It has also been reported that complementary interaction of different groups of non-allelic genes provides stress tolerance. In rice, the complementary interaction of two groups of non-allelic genes (Ic1 and Ic2; Ic3 and Ic4) is responsible for Fe uptake. Ic1 is complementary with Ic3 and Ic2 with Ic4 and shows tolerance to Fe chlorosis (Elango et al., 2022). Overexpression of these genes may also contribute to increasing the phytoremediation efficiency of various plants. Heavy metal ATPases such as HMA3 and HMA4 are also responsible for the accumulation and tolerance of Zn and Cd. Overexpression of heavy metal ATPase 3 (TcHMA3) in *Noccaea caerulea* improved tolerance to Zn and Cd (Sorour et al., 2022).

Another alternative to overcome these challenges is the use of different additives that can help to increase the phytoremediation efficiency of different plants. In one study, different organic fertilizers, including leonardite, bone meal, vermicompost, chicken manure, and bat manure, were employed individually and in combination to test their inoculation effect on phytoremediation efficiency of *Acacia mangium*, *Jatropha curcas*, and *Manihot esculenta*. *Acacia mangium* and *Manihot esculenta* reportedly grew better when treated with bone meal and bat dung and with leonardite and bat dung amendments, respectively, while *Jatropha curcas* reportedly grew better when treated with bone meal, which improved the phytoremediation properties of Cd (Taeprayoon et al., 2022). Biochar can immobilize heavy metals and, therefore, can be used to remediate contaminated soil. Under non-flooded conditions, bamboo biochar boosted Cd and Zn transport from roots to aboveground parts by 68.85% and 102.27%, respectively, compared to no BBC amendment (Li et al., 2022). The use of multi-stress tolerant PGPB with high bioremediation ability against various contaminants in combination with different plants can also be explored.

## 3 PGPB and their role in mitigating abiotic stresses

Agricultural productivity is affected by various environmental stress factors, which are divided into abiotic and biotic stresses. Abiotic stress contributes to a 50% loss in productivity and biotic stress to 30% (Kumar and Verma, 2018). Salinity, drought, flooding, HM contamination, extreme temperatures, and pH are major abiotic stress factors. PGPBs are well-known for their ability to mitigate the negative effects of stress on plants by influencing processes related to their stress response. In the natural environment, plants coexist with various microorganisms (microbiota), belonging to different domains and kingdoms: Archaea, bacteria, fungi, other eukaryotes, and viruses. The rhizosphere provides nutrients and ecological niches for the growth of microbiota, while microorganisms (PGPB) provide beneficial compounds, including phytohormones, which defend plants from diseases and pathogens (Schirawski and Perlin, 2018; Singh et al., 2019; Poria et al., 2021a). The application of PGPB formulations is beneficial for plant development and a way to transform damaged and uncultivable land into healthy soil (Gouda et al., 2018).

### 3.1 PGPB as a salinity tolerant agent

It is estimated that up to 33% of total agricultural land worldwide is salinized (Otlewska et al., 2020). The main mechanism which contributes to salinity tolerance in plants is the translocation of sodium to vacuoles, thus reducing the amount of sodium in the cytoplasm. Research shows that PGPB treatment is associated with increased expression of genes encoding the Salt Overly Sensitive 1 (SOS1) exchanger located in the plasma membrane and other genes connected with the SOS pathway. The main consequence of salinity stress is the increase in the production of phytohormones such as abscisic acid, salicylic acid, and ethylene, which are responsible for activating the signaling cascade of various genes that are involved in enhancing salt tolerance (Ramakrishna et al., 2020). Studies suggest that soil inoculation with specific PGPB strains (*Bacillus aryabhattai* H19-1 and *B. mesonae* H20-5) is linked to increased antioxidant enzyme activity, metabolism of abscisic acid, and proline accumulation, under salinity stress (Sung-Je et al., 2019).

Production of ACC deaminase by PGPB is the best-studied mechanism that contributes to salinity tolerance in plants. Ali et al. (2014) observed that in the presence of PGPB *Pseudomonas* spp., tomato plants under 165 mM of salt stress have shown a 2.5- to 3.5-fold increase in dry weight compared to uninoculated controls and treatments inoculated with *Pseudomonas* spp. mutants that were deficient in ACC deaminase. Another important mechanism to combat salinity stress is the production of exopolysaccharides (EPS) and biofilm formation. Inoculation of chickpea with *Halomonas variabilis* HT1 and *Planococcus rifietoensis* RT4 strains improved fresh weight by 153% and 177%, respectively, under a NaCl concentration of 100 mM (Qurashi and Sabri, 2012).

### 3.2 PGPB as a HM-tolerant agent

Approximately 20 Mha of land around the globe is affected by HM contamination (Liu et al., 2018). HMs occur naturally in the Earth’s crust, and their overuse in industrial production has resulted in the contamination of large areas of land (Liu et al., 2018). In plants and other biological organisms, contact with HMs can affect components of cellular organelles and important enzymes. PGPB can mitigate the effects of exposure to HMs in plants and increase biomass production (Tchounwou et al., 2012; Ma et al., 2016b). The HM-polluted environment can be restored for crop production by either eliminating it or converting it to a non-bioavailable form (Tak et al., 2013).

Microbial populations exposed to HMs develop the resistance for these HMs to survive. These PGPB may become tolerant to HMs due to their biological properties or use direct detoxification and, therefore, become resistant (Ledin, 2000). Such mechanisms developed by PGPB include metal-protein complex formation, biotransformation, methylation, and demethylation (Tak et al., 2013). For instance, *Bacillus thuringiensis* strain GDB - 1 enhanced the metal removal activities of *Alnus firma* by acting on Pb, Zn, As, Cd, Cu, and Ni or reducing their toxicity by accumulating those metals in the seedlings of *Alnus firma* (Babu et al., 2013). Another study showed that maize inoculated with *Proteus mirabilis* T2Cr and CrP450 reduced Cr toxicity and increased Cr tolerance. It was found that the fresh weight in maize increased by 114% compared to the uninoculated control (Islam et al., 2016). In one study, the metal tolerant PGPR *Pseudomonas* and *Bacillus* increased the hyperaccumulation capacity of *Helianthus annuus* L. and caused a 1.7–2.5-fold accumulation of Zn and Cd in *Helianthus annuus* L. shoots (Sorour et al., 2022). The uptake, localization, intracellular transport, and efflux of Zn are regulated by Zn-related transporters, which include the yellow stripe-like (YSL) transporter, the copper transporter (COPT/Ctr), the P1B-type heavy metal ATPase (HMA), the cation efflux (CE) transporter, the Zn- and Fe-regulated transporter-like protein (ZIP), and the Zn-induced facilitator1 (ZIF1) transporter family. ZNTs are zinc-resistant transporters and iron-resistant transporter-like protein (ZIP) family micronutrient transporters. These transporters can be targeted for improving the resistance of plants against heavy metals. Similarly, Arbuscular mycorrhiza fungi (AMF) increased the resistance of *E. grandis* to high-Zn stress by improving nutrient uptake and regulating Zn uptake at the gene transcription level. With AMF symbiosis under high-Zn conditions, 10 genes (ZNT:4, COPT/Ctr:2, YSL:3, CE:1) were upregulated, while 19 genes (ZNT:9, COPT/Ctr:2, YSL:3, ZIFL:4, CE:1) were downregulated (Wang et al., 2022).

### 3.3 PGPB as a drought-tolerant agent

Drought affects more than 160 Mha of rain-fed land used to grow cereals and legumes. Moreover, due to climate change, water deficiency will affect even more lands, as global warming is projected to increase by around 0.2 to 0.5 each decade in Asian regions and by up to 1.6 in South Africa (Berger et al., 2016).

Drought triggers a signaling pathway that accumulates reactive oxygen species (ROS) in plants, which can damage cell membranes, proteins, and DNA in the absence of detoxification mechanisms (Saikia et al., 2018; Yang and Guo, 2018). These mechanisms may be enzymatic involving enzymes such as catalase (CAT), peroxidase (POD), and superoxide dismutase (SOD), or non-enzymatic, relying on antioxidant compounds such as polyphenols and ascorbic acid (Tiepo et al., 2020). Inoculation with PGPB also enhances the antioxidant response of plants. For instance, inoculation of *Urochloa ruziziensis* leaves with *Azosprillum brasilense* increased the content of CAT and POD in plant tissues, which may have contributed to increased drought tolerance (Bulegon et al., 2016). PGPB can also help the plant endure drought stress by secreting exopolysaccharides (Sun et al., 2020) and by ACC deaminase synthesis. In one study, a consortium of three PGPB strains was shown to improve chlorophyll content in drought-stressed black gram plants by more than 100% and garden pea plants by up to 280% (Saikia et al., 2018).

## 4 PGPB’s role in phytoremediation

Phytoremediation is one of the few universal methods that can be used to treat almost all types of marginal land due to the diversity and high remedial ability of different plants (Thijs et al., 2017). However, in some marginal lands, especially with highly toxic contaminants, or other stressed environments, the efficiency of phytoremediation may be lower. One way to improve the effectiveness of phytoremediation is by the employment of microorganisms which can support the growth of plants under such stressed environments and contribute to the mobility of contaminants in soil (Yan et al., 2020). Soil bioaugmentation with an exogenous/endogenous pool of microbes or stimulations of the activities of autochthonic microbiota, which can establish beneficial relationships with plants, can improve the phytoremediation abilities of these plants (Baneshi et al., 2014; Ferrarini et al., 2021; Rabani et al., 2022). The role of different PGPBs in the phytoremediation of several contaminants is illustrated in **Table 2**.

**Table 2:** Depiction of PGPB role in phytoremediation of different pollutants.

Plant name	PGPB	Role of PGPB	Contaminant removed	Reference
*Medicago sativa*	Co-inoculation of *Paenibacillus mucilaginosus* and Cu resistant rhizobia *Sinorhizobium meliloti*	Increased the phytostabilization of Cu and prevented toxic metals from entering the food chain, increased shoot and root biomass, increased total nitrogen, available potassium, and soil organic matter content, microbial community structure, and increased soil enzymatic activity.	Cu	Ju et al. (2020)
*Robinia pseudoacacia* L.	*Enterobacter* sp. YG-14 combined with sludge biochar	Increased Cd stabilization through chelation, increased shoot and root biomass, and biochar amendment increased the survivability of PBPB in harsh environments.	Cd	Zhang et al. (2021)
Corn	*Pseudomonas putida* in combination with EDTA	Microbial inoculation increased phytostabilization efficiency.	Cd, Pb, and Zn	Hamidpour et al. (2020)
*Helianthus petiolaris*	*Bacillus* spp.	Increased shoot biomass and lowered HM uptake.	Pb, Cd	Saran et al. (2020b)
*Zea mays*	*Providencia* sp. and *Proteus mirabilis*	Reduced Cr accumulation in the shoot by reducing Cr translocation from root to shoot.	Cr	Vishnupradeep et al. (2022)
Ryegrass	*Bacillus* spp.	Immobilized Cu and Cd, phosphate solubilization, IAA production, increased root and shoot biomass of plant, and increased stressed mitigation ability.	Cu and Cd	Ke et al. (2021a)
*Lolium perenne*	*Rhodococcus erythropolis* *Rhizobium* sp.	Increased effectiveness of photodegradation through enzyme secretion.	Petroleum Hydrocarbons	Pawlik et al. (2020)
*Phragmites australis*	*Acinetobacter*	Increased plant growth and pyridine degradation.	Pyridine	Karaghool (2022)
*Celosia argentea*	*Bacillus megaterium*	PGPB increased the shoot biomass significantly and increased the shoot Cd extraction amount. It enhanced phytoextraction as well as increased soil enzyme activities of the contaminated soil.	Cd	Yu et al. (2020)
*Triticum aestivum* L.	*Streptomyces pactum*	Increased metal uptake, increased wheat growth, decreased soil pH, and/or increased metal chelation and production of indole acetic acid and siderophores, decreased antioxidant activity and lipid peroxidation in wheat.	Cd, Cu, and Zn	Ali et al. (2021)
*Lolium perenne* L.	*Bacillus* spp.	Increased biomass yield, phytoextraction efficiency, and Cu extraction efficiency. Increased soil Cu bioavailability by secreting siderophores and organic acid.	Cu	Ke et al. (2021b)
*Glycine max* L.	*Kocuria rhizophila*	Enhanced plant biomass by about 38.73% and the accumulation of Cd, Cr, Cu, and Ni.	Cd, Cr, Cu and Ni	Hussain et al. (2019)
*Jatropha curcas*	*Bacillus cereus*	Enhanced metal mobilizing activity and increased plant growth by different PGP activities, such as nitrogen fixation, phosphate solubilization, etc.	Bauxite waste	Narayanan et al. (2021)
*Medicago sativa* L.	*Bacillus subtilis*	PGPB decreased malondialdehyde (MDA) amount and improved activities of plant antioxidant enzymes which led to increased plant biomass. Inoculation also increased Cd bioavailability and Cd removal efficiency.	Cd	Li et al. (2021)
*Arundo donax* L.	*Stenotrophomonas maltophilia* and *Agrobacterium* sp.	Leaf and stem biomass increased. The bioaccumulation factor doubled in the presence of PGPB.	As	Guarino et al. (2020)
*Tagetes erecta* L.	*Klebsiella pneumoniae*	Improved plant growth and increased root length, dry root weight, shoot length, and dry shoot weight in the presence of PGPB. Enhanced relative abundance of Firmicutes and Acidobacteria in the presence of PGPB.	Pyrene	Rajkumari et al. (2021)
*Melia azedarach*	*Serratia marcescens* S2I7	Rhizodegradation increased in the presence of PGPB. PBPB enhanced plant growth.	Benzo (A) pyrene and Cd	Kotoky and Pandey (2020a)
*Zea Mays* L.	*Piriformospora Indica*	PGPB enhanced the degradation of petroleum hydrocarbons in the root zone and increased root and shoot biomass.	Petroleum	Zamani et al. (2018)
*Melia azedarach*	*Bacillus flexus* *Paenibacillus* sp.	Rhizodegradation increased in the presence of PGPB	Benzo (A) pyrene	Kotoky and Pandey (2020b)
*Lepironia articulata*	*Pseudomonas toyotomiensis* *Microbacterium resistens* *Bacillus pumilus*	Enhanced removal of Polycyclic aromatic hydrocarbons.	Polycyclic aromatic hydrocarbons	Al Sbani et al. (2021)

### 4.1 PGPB enhanced plant growth on marginal land under HM contamination conditions

The microbial enhancement of the phytoremediation of marginal soils contaminated with HMs may be the result of (i) promoting the growth of plants (Saleem et al., 2019; Debiec-Andrzejewska et al., 2020; Zainab et al., 2021), (ii) increasing plant tolerance against the metal’s toxicity (Khan and Bano, 2018), (iii) increasing the solubility and mobility of HMs (Ferrarini et al., 2021), or (iv) biotransformation of metals to less toxic chemical compounds (Karthik et al., 2017; Debiec-Andrzejewska et al., 2020). Most PGPBs are characterized by more than one plant growth-promoting property; thus, enhancement in the phytoremediation efficiency of HM-contaminated areas is usually complex and is a result of the synergistic effect of various plant-beneficial properties of bacteria (Ahemad, 2015; Sansinenea, 2019; Debiec-Andrzejewska et al., 2020).

Various phytoremediation strategies are used depending on many factors, such as (i) type of HM contamination (one or many metals), (ii) its concentrations, as well as the (iii) availability of plants and microbes species in the contaminated areas (Ullah et al., 2015). Phytoextraction is a commonly used method for HM-contaminated environments. The efficiency of phytoextraction in highly contaminated areas depends largely on the presence of plants and bacteria with high resistance to HMs (Girolkar et al., 2021). PGPBs seem to be highly beneficial for the phytoextraction process since their presence contributes to the additional increase of plant tolerance to HMs. For example, three bacterial strains resistant to Cu, *Burkholderia cepacia* J62, *Pseudomonas thivervalensis* Y1-3-9, and *Microbacterium oxydans* JYC17, contributed significantly to an increase in the biomass of *Brassica napus* L. and the Cu enrichment in the above-ground part by up to 63.4%, 55.3%, and 63.4%, respectively, compared to uninoculated soil (Ren et al., 2019). In other studies, endophytic and Cd resistance *Pseudomonas fluorescens* Sasm05 promoted the growth of *Sedum alfredi* and significantly increased their ability to accumulate Cd by increasing production of IAA and by the upregulation of the gene expression responsible for the uptake and transport of Cd by plants (Chen et al., 2017a; Wu et al., 2020a). The IAA production by metal-resistant *Pseudomonas libanensis* TR1 and *Pseudomonas reactans* Ph3R3 enhanced plant growth and Cu and Zn accumulation by *Brassica oxyrrhina* under HMs and drought stress (Ma et al., 2016b). Lead-tolerant *Bacillus altitudinis* KP14 enhanced the biomass of *Miscanthus* x *giganteus* (MxG) by up to 77%, due to its ability to solubilize phosphorus and produce IAA, ACC deaminases, ammonia, siderophores, and hydrogen cyanide, as well as its high antifungal activity (Pranaw et al., 2020).

The role of microorganisms in phytoextraction was also confirmed in the case of Ni (Ma et al., 2011; Durand et al., 2016; Chen et al., 2017b), Pb (He et al., 2020; Konkolewska et al., 2020), Cd (He et al., 2020), Mn (Huang et al., 2020; Li et al., 2020), Cr (Ahemad, 2015), and As (Debiec-Andrzejewska et al., 2020). Soil bioaugmentation with nickel-resistant bacterium *Psychrobacter* sp. SRS8 resulted in significant growth promotion of *R. communis* and *H. annuus* and doubled nickel phytoextraction efficiency (Ma et al., 2010). PGPB activity increased Ni bioaccumulation in plant tissues by improving their Ni resistance (Chen et al., 2017b). The improved *Solanum nigrum* growth along with greater tolerance to Pb and Cd stress was observed after inoculation with *Bacillus* sp. QX8 and QX13 (He et al., 2020). Similarly, the inoculation of *Bacillus cereus* HM5 and *B. thuringiensis* HM7 increased the absorption of Mn by *B. papyrifera* by promoting plant root function maintenance and by reducing oxidative stress (Huang et al., 2020). Phytoextraction may also be facilitated by HM stress mitigation for plants by the activity of PGPB (Ahemad, 2015). This mechanism was confirmed in the case of Cr soil contamination (by the microbial Cr(IV)-reducing potential) (Ahemad, 2015) and As (microbial As(III)-oxidizing potential) (Debiec-Andrzejewska et al., 2020).

The choice of HM phytoremediation strategy also depends on the ability of plants to translocate the metal from roots to aboveground parts of plants. This is the so-called translocation factor (TF). Plants species with a TF above 1 are considered a good candidate for phytoextraction, but plants characterized with a TF below 1 are considered appropriate phytostabilizors (Ma et al., 2015). Phytostabilization is usually applied when the HM concentration in soil is relatively low and their potential release to other environments does not present too much risk. This phytoremediation method was used for the stabilization of Zn by *Brassica juncea* and *Ricinus communis* inoculated with *Psychrobacter* sp. SRS8 and *Pseudomonas* sp. A3R3 (Ma et al., 2015). Surprisingly, Fe and Ni in these plants-microbial partnerships were phytoextracted too. Another study revealed that Cd and Zn rhizoaccumulation by *Sedum plumbizincicola* enhanced by E6S strain homologous to *Achromobacter piechaudii* (Ma et al., 2016c). The main mechanism of the microbial enhancement of phytostabilization is the reduced solubility and/or mobility of HMs both in the soil and inside plants. Among these processes are adsorption, biosorption, bioaccumulation, biotransformation, (bio)precipitation and complexation of HMs, and alkalization of soil (Manoj et al., 2020). The key role in adsorption is played by anionic-charged EPS and extracellular membrane functional groups, which can adsorb cationic-charged metals (Wang et al., 2010; Karthik et al., 2017). Biosorption and bioaccumulation processes are directly correlated with passive or active transport to microbial cells, and after that, metals are intracellularly immobilized by precipitation, accumulation, sequestration, and/or transformation (Ma et al., 2016a). Microbes are also able to biotransform HMs, thus decreasing their mobility and bioavailability.

The last commonly used method for the phytoremediation of HM-contaminated lands is phytovolatilization. This method is used in the case of HMs that can be biotransformed to less toxic volatile compounds. Phytovolatilization is, therefore, mainly used to treat lands contaminated with As, Se, and Hg, and the efficiency of this method may be also strengthened by the activity of PGPB. Plant-beneficial strains *Stenotrophomonas maltophilia* and *Agrobacterium* sp. were useful in effective phytovolatilization of As during the investigation of phytoremediation technology with the use of *Arundo donax* L (Yan et al., 2020). The mechanism for As phytovolatilization depends on the As-speciation in soil, but generally, it relies on the intracellular reduction of As(V) to As(III) by plants, the addition of methyl groups to As(III), and then further reduction of the methylated form of As (Chen et al., 2017c). Assisting microorganisms in this process relies on an increase in the As uptake efficiency from the soil, which causes more As to phytovolatilize (Guarino et al., 2020). Microbial enhancement of phytovolatilization was also confirmed in the case of Se contamination. Phytovolatilization relies on the transformation of toxic Se (as selenate) to the less toxic dimethyl selenide gas (Pivetz, 2001). An example of the enhanced Se phytovolatilization was the activity of environmental isolates BJ2 and BJ15, which supported the uptake and evaporation of selenium by *Brassica juncea* L. It was shown that bacteria contributed to the high efficiency of volatilization and accumulation of Se in plant tissues, which were enhanced by 35% and 70%, respectively (De Souza et al., 1999). The microbial enhancement of Hg phytovolatilization efficiency is based on decreasing the toxicity of this element by reduction of organomercurials to Hg(II) (Matsui et al., 2016) or Hg(II) to less toxic and volatile Hg(0) (Silver and Hobman, 2007). *Zea mays* inoculation by endophytic bacteria *Serratia marcescens* BacI56 and *Pseudomonas* sp. BacI38 increased Hg volatilization by 47.16% and 62.42%, respectively (Mello et al., 2020). Despite the confirmed cases of microbial-assisted Hg phytovolatilization, knowledge about molecular and physiological aspects of this mechanism is still poor (Tiodar et al., 2021).

### 4.2 PGPB-enhanced plant growth on marginal land with high salinity

Marginal land can have high salt concentration due to a variety of reasons, and sources of this high salinity can be natural resulting from (i) salt accumulation over a long time, (ii) weathering of rocks and minerals that release soluble salts including chloride, sulfates, and carbonates of sodium, calcium, and magnesium, or (iii) deposition of oceanic salt carried by wind and rain (Mukhopadhyay and Maiti, 2010). Anthropogenic sources are the result of the (i) replacement of perennial vegetation with annual crops, (ii) inappropriate fertilization or soil irrigation with salt-rich water, or (iii) insufficient drainage systems (Mukhopadhyay and Maiti, 2010). High salinity in soil contributes to ion imbalance and hyperosmotic pressure, leading to oxidative stress in plants (Heydarian et al., 2016). High salt concentrations may also decrease the availability of essential nutrients in the soil which leads to a reduced uptake of some nutrients by the plants (Pankaj et al., 2019). High salinity environments have been shown to elevate ethylene concentration, which is a typical reaction of plants under stress conditions (Heydarian et al., 2016). PGPB helps plants to alleviate salinity stress in marginal lands by reducing ethylene precursors through the production of ACC-deaminase (Ali et al., 2014; Sofy et al., 2021) and the production of biofilms covering the surface of the roots, limiting the physical contact of roots with contaminants (Kasim et al., 2016). PGPB also increases water use efficiency by transpiration regulation, stomatal conductance, and reduction of reactive oxygen species concentrations in inoculated plants (Amaresan et al., 2020).

Application of ACC deaminase-producing *Pseudomonas* spp. on barley and oat for phytoremediation of marginal lands with high salinity resulted in promotion of the growth of barley and oats roots in saline soil by 200% and 50%, respectively, and contributed to an increase in the shoot’s biomass by 100%–150%. This significantly increased plant biomass was able to accumulate higher amounts of salt (Chang et al., 2014). Similarly, *Pseudomonas putida* KT2440 and *Novosphingobium* sp. HR1a promoted the growth of *Citrus macrophylla*. The strain HR1a contributed to increased accumulation of IAA in leaves, while the strain KT2440 inhibited root chloride and proline accumulation under salt stress (Vives-Peris et al., 2020). Spinach (*Spinacia oleracea* L) inoculated with halotolerant (*Pseudomonas* sp., *Thalassobacillus* sp., and *Terribacillus* sp.) and chitinolytic (*Pseudomonas* spp., *Sanguibacter* spp., *Bacillus* spp.) bacterial strains with high antifungal activity resulted in better plant growth and reduced salinity (Anees et al., 2020).

### 4.3 PGPB enhanced plant growth on marginal lands contaminated with organic compounds

Due to the strong hydrophobic characteristics of many organic contaminants, phytoextraction efficiency is usually lower than that of phytodegradation or phytostimulation (Schwitzguébel, 2017). Bacteria use the metabolites secreted by the plant as carbon sources to stimulate their biodegradation ability, which, in turn, reduces the stress effect of the contaminant and promotes the growth and development of plants (Girolkar et al., 2021).

Among PGPB useful in phytostimulation are highly resistant microbes to hydrocarbons, which can degrade organic contaminants aerobically. Particularly desirable are strains capable of degrading crude oil hydrocarbon, the most pervasive class of environmental organic contaminants worldwide (Ławniczak et al., 2020). The source of PGPB in phytostimulation methods is usually autochthonic microbiota, whose activity is stimulated by plants. Soil bioaugmentation with an exogenous pool of microorganisms as well as combined methods is also applied (Schwitzguébel, 2017; Girolkar et al., 2021).

*Acinetobacter*, *Alcanivorax*, *Bacillus*, *Corynebacterium*, *Gordonia*, *Hahella*, *Immundisolibacter*, *Luteimonas*, *Marinobacter*, *Mycobacterium*, *Ochrobactrum*, *Pseudomonas*, *Rhodococcus*, and *Sphingomonas* are some of the reported genera capable of degrading organic pollutants effectively (Kukla et al., 2014; Oliveira et al., 2014; Fatima et al., 2015; Pawlik et al., 2017). Despite the high diversity of these strains, the rhizosphere microorganisms are regarded as the most effective in hydrocarbon degradation (Ruley et al., 2020). Biosurfactant production by these bacterial strains helps in increasing the bioavailability of petroleum compounds, which plays an important role in biodegradation (Ławniczak et al., 2020).

The positive effect of PGPB was observed in the phytoremediation of Ni-pyrene-contaminated soil with the use of *Scripus triqueter* (Chen et al., 2017b). Although PGPB presence decreased the plant’s biomass, their activity facilitated the increase in the plant’s resistance to pyrene and promotion of pyrene degradation (Chen et al., 2017b). High efficiency of the phytostimulation was achieved in crude petroleum oil-contaminated soil with the use of *Bassia scoparia* supported by associated rhizosphere microorganisms (Moubasher et al., 2015). A similar effect of PGPB was observed in the case of petroleum hydrocarbon degradation by Italian ryegrass (Hussain et al., 2018), petroleum oily sludge phytoremediation with the use of legumes plant *Cajanus cajan*, and rhizospheric microorganisms (Allamin et al., 2020), and diesel contaminant degradation by *Zea mays* L. supported by alkane‐degrading bacterial strains (Ummara et al., 2021).

Phytostimulation may also be used as an additional strategy for the remediation of organic compounds on contaminated lands. For example, in the polychlorinated biphenyl (PCB) removal by switchgrass (*Panicum virgatum*), phytostimulation with the use of plant-beneficial bacteria *Burkholderia xenovorans* LB400 was an additional strategy to the main treatment mechanism – phytoextraction. In this case, a quite effective translocation of PCB was confirmed, but enhanced microbial activity in the rhizosphere was also observed (Liang et al., 2014).

Numerous biotechnological strategies that promote plant tolerance and metal accumulation, such as the modification of HM transporter genes and their uptake systems, along with the improvement of HM ligand production, can also be used to improve phytoremediation (Bhat et al., 2022). Overexpression of HM transporter gene in *Arabidopsis thaliana* (YCF1 gene) improved tolerance to and accumulation of Pb and Cd. Similarly, transgenic plants that overexpressed the *Nicotiana tabacum* NtCBP4 protein displayed enhanced Pb hypersensitivity and accumulation. HM-binding ligands, phytochelatins, glutathione, and cystine-rich peptides, such as metallothioneins, are utilized for HM detoxification. In peas, due to the overexpression of the metallothioneins gene PsMTA, significant Cu buildup in roots was detected. Similarly, transgenic *Brassica juncea* with overexpression of *E. coli* GSH synthetase gene showed improved Cd tolerance (Bhat et al., 2022).

## 5 Economic perspectives of bioremediation and marginal land utilization for biomass production

The global bioremediation market was valued at USD 105.68 billion in 2019 and is expected to grow at a compound annual growth rate (CAGR) of 15.5% to reach USD 334.70 billion by 2027. In light of contemporary green technologies to improve waste management, awareness of bioremediation is growing. Due to their affordability and ease of use, microbiological counterparts to bioremediation are being utilized more frequently than other environmentally friendly techniques. Growing urbanization causes land to become marginalized, or less suited for agricultural, in many regions of industrialized and developing countries. The phytoremediation segment held the majority of the market share in 2019. Plants are extensively used to kickstart the process and are driving the growth of the segment. In terms of market share, North America accounted for approximately 41.8% of the global bioremediation market in 2019. The global microbial bioremediation market is quite fragmented, with a few large and medium-sized market players accounting for most of the revenue. The major players are implementing a variety of strategies, including mergers and acquisitions, strategic agreements and contracts, and the development, testing, and deployment of more effective microbial bioremediation systems. As governments across the globe are trying to attempt to focus on environmental protection, the market for bioremediation technology and services is expected to grow significantly during the forecast period. The bioremediation market is expected to reach new heights by 2030. This is due to the above-mentioned factors as well as the tremendous growth in research and development activities throughout the world (Emergen Research, 2022).

The global microbial bioremediation market is segmented into bacteria, fungi, and archaea. Bacteria are the most used organisms in the bioremediation process. **Table 3** shows the United States Environmental Protection Agency’s national contingency plan for planned bioremediation products (EPA, 2022).

**Table 3:** Commercial microbial inoculants (culture formulation) for bioremediation in soil.

Tradename	Manufacturer	Product form	Application method	Type of contamination	Shelf life
BET BIOPETRO	BioEnviro Tech	Powder	–	Heavy refined and crude hydrocarbon contaminants	More than 3 years
BIOREM-2000 OIL DIGESTER™	Clift Industries, Inc., Charlotte, US	–	Spray	Oil	2 years
BioWorld BHTP	BioWorld USA, Inc., US	Liquid or dry form	Directly into the soil or mix with water before use	Hydrocarbons	Max 3 years
DRYLET™ MB BIOREMEDIATION	DryLet, LLC, Houston, US	–	Applied by the usual methods of aerial or manual broadcast spreading	Oil	Min 5 years
DUALZORB^®^	LBI Renewable, Buffalo, US	Dehydrated product	Applied by hand, mechanical spreaders, portable mixer or blown onto a surface using an air conveyor	Hydrocarbons	5 years
ERGOFIT MICROMIX AQUA	Evadine Technologies, LLC, New Braunfels, US	Liquid	Spray	Hydrocarbons	5 years
MICRO-BLAZE EMERGENCY LIQUID SPILL CONTROL	Verde Environmental, Inc., Houston, US	Liquid formulation	Spray	Organics and hydrocarbons in soil and water as well as control odors	Min 10 years
MUNOX SR^®^	Osprey Biotechnics, Sarasota, US	Stabilized liquid form or after the freeze-dried form is hydrated	Spray	Fresh and weathered crude oil and refined products	1 year
OPPENHEIMER FORMULA (The OPPENHEIMER FORMULA I, MICROSORB SC)	Oppenheimer Biotechnology, Inc., Pflugerville, US	Powder seeding	Spray	Oil	5 years
SOIL RX	3 Tier Technologies LLC Worldwide Headquarters, Stanford, US	Liquid	Sprayed after dilution	Organics and hydrocarbons	2 years
STEP ONE	B & S Research, Inc., Embarrass, US	–	Spray	Most hydrocarbons, including crude and refined petroleum products, pesticides	Over 3 years
SYSTEM E.T. 20	Environmental Restoration Services, Windsor, US	–	–	Broad range of hydrocarbon compounds	2 years
WMI-2000	WMI International, Inc., Houston, US	–	Spray	–	2 years

Another economic aspect of bioremediation is the use of contaminated soil for the production of industrially important raw materials such as biomass while carrying out remediation. Global economic growth and industrialization require a continuous supply of energy, which dramatically decreases natural energy resources. Therefore, an intense search for alternative and renewable energy sources to meet the demands of an ever-evolving world is underway (Mehmood et al., 2017). The latest estimates show that about 19% of global energy demand is met by renewable sources, of which biomass accounts for 9.3% (Edrisi and Abhilash, 2016). Among the renewable energy sources currently available, plant biomass is regarded as one of the most promising (Mehmood et al., 2017). In comparison to fossil fuels, the primary benefit of biomass as an energy source is its beneficial effect on the global CO_2_ balance in the atmosphere, since more CO_2_ is absorbed by plants during photosynthesis than is emitted to the atmosphere during biofuel combustion (Baumgarten et al., 2017). Bioenergy derived from a plant’s biomass also has a considerable influence on the mitigation of various environmental problems; e.g., high-yielding plants may be used for the phytoremediation of contaminated soils. Thus, bioenergy plant cultures may constitute an additional possibility for marginal land restoration (Edrisi and Abhilash, 2016).

The availability of land for biofuel production is limited (arable lands cannot be considered due to the high demand for food production). More and more frequently, the utilization of marginal and degraded lands is a viable solution to meet the energy needs of the people (Pancaldi and Trindade, 2020). The total world surface area of marginal lands is estimated at 13.1 Gha, of which 152 Mha (14% of the total marginal land area in the world) are available only in China. It is also estimate, that out of this 152 Mha, 60% may be utilized for bioenergy crop plantation. The current bioenergy potential of plants growing on marginal lands may range from 30 to 1000 Exa-Joule (EJ; 1 EJ=1018 J) of primary energy per year (Haberl et al., 2010). Considering the imperatives of sustainable economic development, it is estimated that the bio-energy potential by 2050 will be in the range of 130 - 270 EJ per year (Hoogwijk et al., 2009).

Several bioenergy crops are characterized by having high resistance to stressful environmental conditions and achieving high biomass in poor soils without special fertilization or other agricultural inputs (Hood, 2016). The most promising energy crop is *Miscanthus* species, which is regarded as a second-generation biofuel plant capable of producing high amounts of biomass due to effective photosynthesis and high water and nutrient usage efficiencies (Lewandowski et al., 2019; Pidlisnyuk et al., 2020). Plants such as *Agave* spp., *Cynara cardunculus* (cardoon), *Panicum virgatum* L. (switchgrass), *Phalaris arundinacea* L. (reed canarygrass), *Sida hermaphrodita* (sida), *Ulmus pumila* (Siberian elm), *Sorghum bicolor* L. (sweet sorghum), and *Salix* spp. (willow) are also considered good candidates for bioenergy crops (Saballos, 2008; Soldatos, 2015; Mehmood et al., 2017). Some of them are more adaptive to changing environmental conditions, but all of them have shown high potential for effective biomass production (Soldatos, 2015; Mehmood et al., 2017). **Table 4** shows some important bioenergy crops and their yields.

**Table 4:** Some important bioenergy crops with their yield in contaminated land.

Bioenergy crop	Contaminants removed	Yield	Reference
*Helianthus annuus* and *Zea mays*	Cr, Cu, Pb, and Cd	84-87% increase in biomass	Iram et al. (2019)
*Manihot esculenta*	Cu, Pb, and Zn	Fresh tuber yield of 23.13–26.22 t/ha	Shen et al. (2020)
Canola, oat, and wheat	Cd	159.37%, 179.23% and 111.34% increase in biomass	Zhang et al. (2013)
*Phragmites australis*, *Arundo donax*, and *Piptatherum miliaceum*	Zn, As, Cd, and Pb	Heating values of biomass 16.03–18.75 MJ/Kg	Bernal et al. (2021)
*Miscanthus* × *giganteus*	Cd and Hg	6.3–13.9 t/ha	Zgorelec et al. (2020)
*Brassica napus* L.	Cd	109 g/plant	Wu et al. (2020b)
*Lantana camara* L.	Cd	12.8 g/pot	Liu et al. (2019)
*Brassica juncea* L.	Cd	14.4 g/pot	Dhaliwal et al. (2020)
*Lolium multiflorum* Lam	Cd	1656.6 kg/km^2^	Hu et al. (2020)
*Miscanthus floridulus*	Pb	22.4 t/ha	Cheng et al. (2016)
*Sorghum bicolor* L.	Cd	27.9 t/ha	Xiao et al. (2021)

Despite the high potential and diversity of bioenergy crops, the cost of cultivation and biofuel production is one of the most important factors in deciding their successful commercialization (Mehmood et al., 2017). Current studies indicate that bioenergy produced from the plant’s biomass is still more costly than fossil fuel production; e.g., the cost of cellulosic ethanol is three times higher than the current price of gasoline on an energy equivalent basis (Carriquiry et al., 2011).

Economic analysis showed that the profitability of the cultivation of *Miscanthus* in Europe and the USA depended largely on uncertain assumptions such as environmental factors. Depending on environmental conditions like the amount of rainfall or temperature, *Miscanthus* yield may vary from 10–48 t dry matter per ha. The significant differences in yield have a direct influence on biomass prices, which may vary in the range of 48–134 €/t dry matter, and thus on the price of bioenergy, which increases in direct proportion. Depending on the species and environmental conditions, the lifespan of plants plays an important role in the profitability of *Miscanthus* cultures and is estimated at 10–20 years (Witzel and Finger, 2016).

In terms of cost, biomass production can depend on many factors, including the species of cultivated plants. For example, in the case of switchgrass, the biomass production cost ranged from $40–61 Mg to $53–74 Mg in Tennessee and Oklahoma in the USA, respectively. Furthermore, the target cost for the commercial production of alcohols from biomass was estimated at US$ 38 Mg. Modeling studies on the commercialization of switchgrass found that the US$ 11–27 Mg expenditure on switchgrass biomass production is justifiable if losses due to sedimentation and soil erosion caused by plant roots are reduced (Mehmood et al., 2017). Another example showed that the cost of the production of biomass in the case of giant reed was up to US$85 per ton, and this species was regarded as the most profitable among two other bioenergy plants, *Miscanthus* and switchgrass (Soldatos, 2015).

There are many barriers to growing energy crops on marginal lands. These include (i) the lack of solid and established markets for trade, (ii) the high cost of crop establishment and uncertainty about crop quality, (iii) unstable prices over the long term (Saballos, 2008; Soldatos, 2015; Mehmood et al., 2017; Pancaldi and Trindade, 2020). Farmers can receive direct or indirect support in the form of subsidies to make the cultivation of energy crops on marginal lands more lucrative in the long run. Unfortunately, the current level of support varies widely across countries and is often insufficient (Mehmood et al., 2017). The cultivation of energy crops on marginal lands to produce alternative fuels is a highly promising option keeping in view the increasing demand for fuel and the decline in arable land. To fully leverage the potential of bioenergy crops, we need to better understand the environmental impact of large-scale production of energy crops using marginal lands worldwide.

Two aspects influence the economics of phytoremediation: application potential and cost compared to traditional treatments (Wan et al., 2016). Using conventional methods involving excavation and filling or ex-situ cleaning of the soil would cost approximately €280,000 and € 680,000 per hectare, respectively, compared with the economic value of phytoremediation of €14,850 and €14,600 per hectare, respectively, as determined by hedonic price analysis and substitution costs. The conventional method generally reduces soil fertility because soil is either landfilled or washed away during the cleaning process (Lewandowski et al., 2006). The plants used for phytoremediation can be used for metal recovery, bioenergy production, packaging materials, and house and furniture construction. However, some phytoremediation techniques are not able to remove the contaminants from soil, such as phytostabilization, and in most of these techniques, the concentration of contaminants in the plant exceeds critical limits so that they can enter the food chain (Parveen et al., 2022). Therefore, contaminants such as HMs can be extracted from plants after remediation using various industrial techniques (phytomining), and high profits can be made due to the costly nature of certain HMs (Riaz et al., 2022).

## 6 Future research directions

Plants used in bioremediation can provide several benefits, including efficient utilization of polluted biomass to produce various value-added products like pigments, platform chemicals, etc., and energy production through cogeneration. Moreover, metals recovered from plant combustion can be utilized as raw materials in industrial processes. Several future areas of research are waiting to be explored. First is estimating the influence of various contaminants on each other’s dynamics and their toxicity to soil microbial populations in the presence of selective plant species such as *Miscanthus* sp. The use of genetically modified plants may provide additional benefits, but their use should be carefully considered on a case-by-case basis and not generalized. Special care should be taken to explore genetically modified bacteria supporting phytoremediation and improving soil health, organic carbon dynamics, and soil biodiversity. This allows the use of plants that previously could not be used in contaminated soils due to their high sensitivity to a particular metal. This is especially important with regard to plants that have other outstanding properties, such as very rapid growth. So far, the impact of climatic change on the dynamics of various contaminants in plants and associated microbial metabolites has been overlooked.

## 7 Conclusion

Plants and bacteria both provide phytoremediation of hazardous pollutants, but bacteria also protect plants from a variety of stresses (HM toxicity, osmotic stress, salinity stress, etc.) and promote plant growth through numerous PGP processes (hormones and siderophores secretion, mineral solubilization, nitrogen fixation, etc.). Despite decades of research, phytoremediation is still considered a relatively new option because there are few long-term field trials. Microorganisms play an important role in the phytoremediation of soils by directly degrading or biotransforming contaminants and indirectly improving the growth of phytoremediated plants. In particular, the study of bacteria that have evolved to be resistant to high metal/pollutant concentrations and their host linkages can add a new dimension to existing phytoremediation techniques. Soil inoculation with PGPB and metal-solubilizing microbes show a lot of potential for boosting hyperaccumulators’ health, biomass yield, and metal accumulation capabilities. Further research is needed on the effectiveness of microbe-assisted phytoremediation under abiotic stresses such as climate change-induced drought and salinity. Furthermore, more research is needed to better understand the interactions between microbes and their host plants in metal-contaminated ecosystems.

## Author contributions

Conceptualization, KP. Writing—original draft preparation, VP, KD-A, AF, ML, and NA. Writing—review and editing, KP and SS. Supervision, KP. All authors have read and agreed to the published version of the manuscript.

## Funding

This research was funded by the grant from the project “The Fly Ash as the Precursors of Functionalized Materials for Applications in Environmental Engineering, Civil Engineering and Agriculture” (no. POIR.04.04.00-00-14E6/18-00), carried out within the TEAM-NET programme of the Foundation for Polish Science co-financed by the European Union under the European regional development fund.

## Acknowledgments

VP acknowledges the DBT-JRF fellowship (award letter no. DBTHRDPMU/JRF/BET-22/I/2022-23/45) provided by the Department of Biotechnology, Government of India.

## Conflict of interest

The authors declare that the research was conducted in the absence of any commercial or financial relationships that could be construed as a potential conflict of interest.

## Publisher’s note

All claims expressed in this article are solely those of the authors and do not necessarily represent those of their affiliated organizations, or those of the publisher, the editors and the reviewers. Any product that may be evaluated in this article, or claim that may be made by its manufacturer, is not guaranteed or endorsed by the publisher.
